# Signaling by two-component system noncognate partners promotes intrinsic tolerance to polymyxin B in uropathogenic *Escherichia coli*

**DOI:** 10.1126/scisignal.aag1775

**Published:** 2017-01-10

**Authors:** Kirsten R. Guckes, Erin J. Breland, Ellisa W. Zhang, Sarah C. Hanks, Navleen K. Gill, Holly M. S. Algood, Jonathan E. Schmitz, Charles W. Stratton, Maria Hadjifrangiskou

**Affiliations:** 1 Department of Pathology, Microbiology and Immunology, Vanderbilt University Medical Center, Nashville, TN 37232, USA; 2 Department of Pharmacology, Vanderbilt University School of Medicine, Nashville, TN 37232, USA; 3 Barnard College, New York, NY 10027, USA; 4 Vanderbilt University, Nashville, TN 37240, USA; 5 Department of Medicine, Vanderbilt University Medical Center, Nashville, TN 37232, USA; 6 Veterans Affairs Tennessee Valley Healthcare Services, Nashville, TN 37212, USA; 7 Department of Urologic Surgery, Vanderbilt University Medical Center, Nashville, TN 37232, USA

## Abstract

Bacteria use two-component systems (TCSs) to react appropriately to environmental stimuli. Typical TCSs comprise a sensor histidine kinase that acts as a receptor coupled to a partner response regulator that coordinates changes in bacterial behavior, often through its activity as a transcriptional regulator. TCS interactions are typically confined to cognate pairs of histidine kinases and response regulators. We describe two distinct TCSs in uropathogenic *Escherichia coli* (UPEC) that interact to mediate a response to ferric iron. The PmrAB and QseBC TCSs were both required for proper transcriptional response to ferric iron. Ferric iron induced the histidine kinase PmrB to phosphotransfer to both its cognate response regulator PmrA and the noncognate response regulator QseB, leading to transcriptional responses coordinated by both regulators. Pretreatment of the UPEC strain UTI89 with ferric iron led to increased resistance to polymyxin B that required both PmrA and QseB. Similarly, pretreatment of several UPEC isolates with ferric iron increased tolerance to polymyxin B. This study defines physiologically relevant cross-talk between TCSs in a bacterial pathogen and provides a potential mechanism for antibiotic resistance of some strains of UPEC.

## Introduction

Bacteria occupy diverse niches, either in a planktonic state or in association with a host organism. Thus, constant sampling of the local environment is imperative to ensure bacterial survival and proliferation. Among the signaling networks that bacteria use, the most predominant are two-component systems (TCSs) (1) that comprise a membrane-embedded histidine kinase and a cytoplasmic response regulator. In all reported cases, TCSs become activated in response to specific signals, which may act as ligands that bind to the receptor histidine kinase (2, 3) or act through as-yet uncharacterized mechanisms that activate the receptor (4–7). Upon signal detection, the histidine kinase dimer auto-phosphorylates at a conserved histidine residue located in the cytoplasmic portion of the protein (8, 9). The histidine kinase then transduces the signal by catalyzing the transfer of this phosphoryl group to a conserved aspartate residue on its cognate response regulator (8, 10, 11). Phosphorylation of the response regulator typically results in a conformational change, leading to dimerization and a change in the activity of the response regulator (8, 9, 12–14). Most response regulators mediate output by acting as transcription factors (8, 9, 12). Thus, in response to an incoming stimulus, there is a rapid phosphotransfer event, leading to maximal phosphorylation of the cognate response regulator within 5 to 10 min. For TCSs that autoregulate the expression of the genes that encode them, the phosphorylated response regulator can act as an activator of transcription, and this series of events is referred to as an activation surge (15, 16). In contrast to mammalian kinases, bacterial histidine kinases can be bifunctional, exhibiting both kinase and phosphatase activities toward the cognate response regulator (8, 14). The inherent phosphatase function of histidine kinases prevents aberrant activation by noncognate histidine kinases or other phospho-donor molecules, which in turn ensures proper regulation of downstream targets.

Few examples of TCS noncognate partner interactions have been described in the literature (17–21). A review by Laub and Goulian has categorized nonpartner TCS interactions as beneficial or detrimental to the bacterium, defining these as cross-regulation or cross-talk, respectively (22). In all reported cases of detrimental cross-talk to date, the noncognate interaction occurred in the absence of one cognate partner (23–28). However, there are a few examples of in vivo cross-regulation that occur in wild-type systems (17–19).

Previous studies in uropathogenic *Escherichia coli* (UPEC) determined that in the absence of the histidine kinase QseC, a component of the QseBC TCS, the noncognate histidine kinase PmrB aberrantly phosphorylates QseB, the response regulator of the QseBC TCS (24). In *Salmonella*, PmrB binding to ferric iron promotes the expression of genes involved in lipopolysaccharide (LPS) modifications (29, 30). Other reported activators of PmrB include aluminum(III) and mildly acidic pH (29, 31). Low concentrations of magnesium as well as polymyxin B (PMB) and other antimicrobial peptides activate PmrAB indirectly through the PhoPQ TCS (32–34). Previous studies indicated that QseC kinase activity is enhanced in response to epinephrine, norepinephrine, and autoinducer 3, a secreted bacterial signaling molecule of unknown structure (35).

Deletion of *qseC* in UPEC leads to deregulation of gene expression and attenuation of virulence presumably due to an accumulation of phosphorylated QseB (QseB∼P) as a result of cross-talk from PmrB (23, 36). Consequently, deletion of the *pmrB* gene in the *qseC* deletion mutant suppresses all the adverse phenotypes associated with the absence of QseC (24, 36–38), leading to the hypothesis that QseC phosphatase function is critical to the ability of this sensor to control QseB. In previous studies, we discovered that PmrA, the cognate response regulator for PmrB in the PmrAB TCS, directly bound the *qseBC* promoter and that both PmrA and QseB were required for returning *qseBC* to uninduced transcript abundance 18 hours after exposure to ferric iron (23). Ferric iron is a well-characterized signal that acts as a proxy for various cations that bacteria may encounter within the host or during infection (39). These findings strongly suggested a physiological link between the PmrAB and QseBC systems.

Here, we present evidence of beneficial TCS cross-regulation occurring in pathogenic *E. coli*, where the response to ferric iron is transduced through a single his-tidine kinase, PmrB, but is orchestrated by two response regulators, PmrA and QseB, to mediate resistance to cationic polypeptides like PMB. Biochemical analyses demonstrated that PmrB kinase activity was enhanced toward both its cognate (PmrA) and noncognate (QseB) response regulators in the presence of ferric iron, leading to an activation surge in transcription of the *qseBC* locus that was dependent on both PmrA and QseB. Both the QseB and PmrA response regulators mediated optimal transcription of downstream target genes in response to ferric iron, and this response occurred in a PmrB-dependent manner. These data describe a unique example in which activation of a single bacterial receptor (PmrB) leads to the phosphorylation of both a cognate and a noncognate response regulator to elicit a physiologically relevant response.

## Results

### All components of the QseBC and PmrAB TCSs are required for proper response to ferric iron

**Fig. 1:**
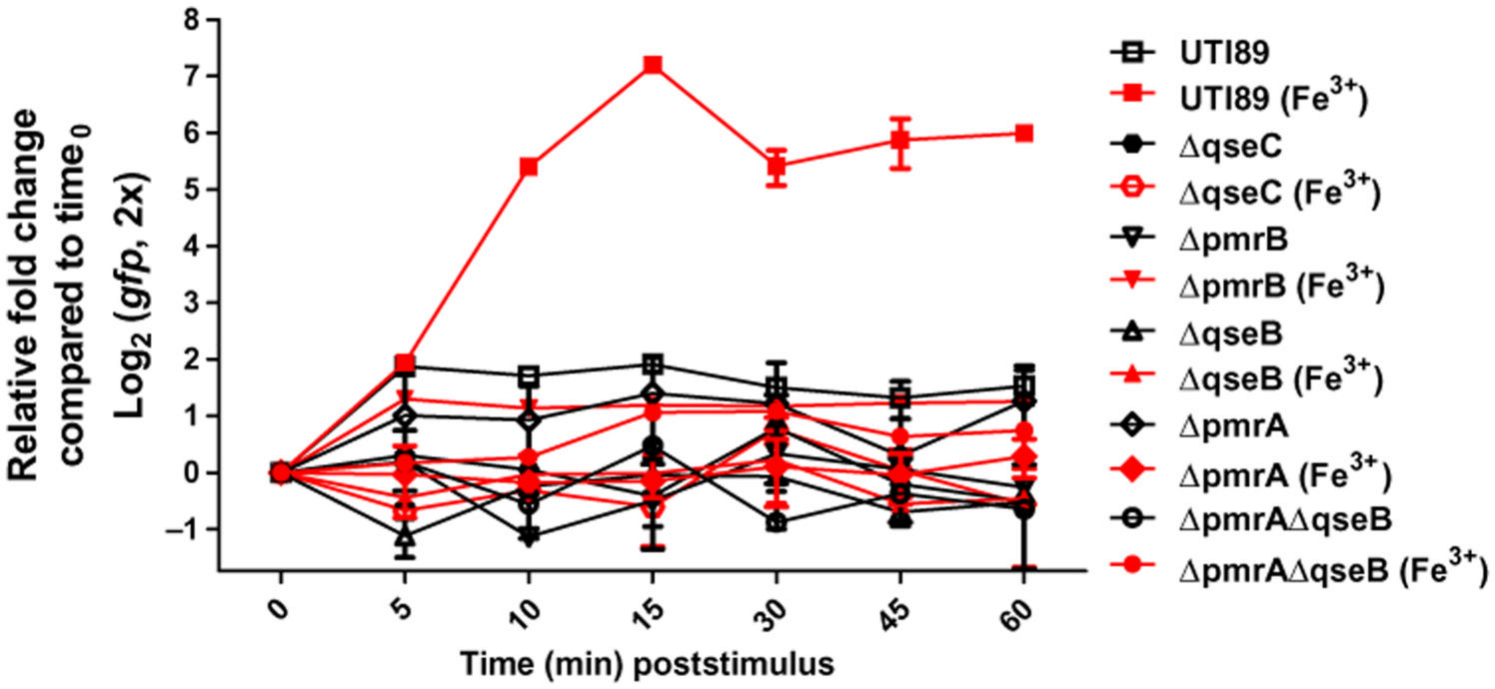
All components of both PmrAB and QseBC are required for the *qseBC* transcriptional surge in response to ferric iron The abundance of green fluorescent protein (*gfp*) transcripts from the *Pqse*∷*gfp* fusion construct in UTI89 and in UTI89 deletion strains lacking components of the PmrAB and QseBC TCSs was determined by qRT-PCR analysis. Analysis was performed both in the absence and in the presence of iron (Fe^3+^). Fold changes were calculated using the ΔΔC_T_ method, with *rrsH* as an endogenous control, and samples were normalized to time 0. Error bars indicate SEM, *n* ≥ 3 for each mutant strain.

Signal reception by bacterial sensor histidine kinases typically leads to an increase in phosphorylation of the response regulator, which in turn alters the expression of target genes within a very short time frame (15). These changes in transcription over time can be followed using reverse transcription quantitative real-time polymerase chain reaction (qRT-PCR) analysis of known target genes (15). We have previously reported that, in UPEC, stimulation with ferric iron induces the *qseBC* operon in a manner that appears to involve the response regulators PmrA and QseB of the PmrAB and QseBC TCSs, respectively (23). To better define this transcriptional control, we measured the activity of the *qseBC* promoter immediately before and immediately after the addition of ferric iron in the clinically isolated UPEC strain UTI89 (40) and in various isogenic UTI89 *pmr* and *qse* mutants using qRT-PCR. Samples of bacteria grown in N-minimal medium were obtained at various time points from 0 to 60 min after exposure to ferric iron, and qRT-PCR analysis was performed to measure the transcriptional surge of the *qseBC* promoter over time (Fig. 1). In the UPEC strain UTI89, a robust surge in steady-state transcript was observed at 15 min after the addition of iron (Fig. 1). These results corroborated previous reports tracking the transcription of PmrA-regulated targets in *Salmonella* (15). However, contrary to what has been reported for *Salmonella* (41), the absence of QseB, QseC, PmrB, or PmrA completely abolished the transcriptional spike seen at 15 min after the addition of iron (Fig. 1). These data implied that in the case of UPEC, all components of both the QseBC and PmrAB TCSs are required to drive the expression of *qseBC* in response to the ferric iron stimulus.

We have demonstrated that in the absence of signal, QseC readily autophosphorylates and phosphotransfers to QseB (fig. S1A) (23, 24). In vitro phosphotransfer assays indicated that the presence of epinephrine (fig. S1A), norepinephrine, or spent UPEC supernatant fractions (42) did not increase the rate of QseC-mediated phosphotransfer under the conditions tested. On the basis of the quantification of QseB∼P (fig. S1, A and B), it appears that epinephrine either decreases the rate of phosphotransfer to QseBor increases the rate of dephosphorylation of QseB by QseC (fig. S1, A to C). Subsequent qRT-PCR analyses probing for changes in *qseB* transcript abundance in the presence of epinephrine indicated no differences in *qseB* steady-state expression in the presence or absence of epinephrine in the UPEC strain UTI89 or in UTI89 lacking the *qseC* gene (fig. S1). The *qseC* deletion mutant UTI89Δ*qseC* showed constitutively high amounts of *qseB* expression, which is the result of unregulated PmrB phosphotransfer to QseB (23). Together, these results indicated that, in UPEC, epinephrine and norepinephrine did not stimulate the kinase activity of the QseC histidine kinase and that QseBC was involved in proper stimulus response to ferric iron in conjunction with PmrAB.

We then probed whether the activation surge we observed (Fig. 1) was specific to stimulation with ferric iron or whether other cations would elicit the same response. To test the specificity of the coordinated response to ferric iron, we used zinc chloride and copper sulfate as sources of zinc (Zn^2+^) and copper (Cu^2+^). These metal cations were chosen on the basis of previous studies identifying high concentrations of extracellular zinc(II) as a putative signal for *E. coli* PmrB (43) and hypersensitivity to toxic ions, such as cesium, cobalt, copper, nickel, and ruthenium, in *E. coli* strains lacking QseBC (44). We tested the steady-state transcript abundance of *qseB* using a qRT-PCR approach similar to that which we used to measure the responses to ferric iron (fig. S2). Only the presence of ferric iron resulted in a typical transcriptional surge (Fig. 1), whereas zinc cations caused a modest and consistent increase in the abundance of *qseB* transcripts, which was maintained over time and did not return to baseline (fig. S2). Addition of copper cations steadily increased the amount of *qseB* transcript over time, reaching maximal transcription at 60 min after stimulation (fig. S2), suggesting that increased qseBC transcription in response to copper may be due to a different copper-responsive regulator and not QseB. On the basis of these observations, we evaluated protein-protein interactions and downstream regulatory events in response to ferric iron.

### PmrB phosphotransfers to PmrA and QseB upon stimulation with ferric iron

**Fig. 2:**
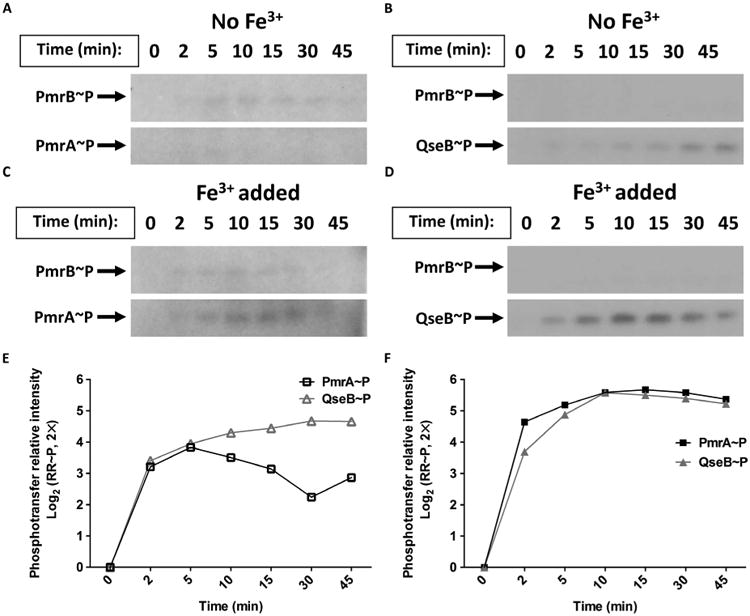
Ferric iron enhances PmrB phosphotransfer activity (**A** and **B**) Representative radiographs tracking the autophosphorylation of PmrB in membrane fractions from UTI89 cells and subsequent phosphotransfer of radiolabeled adenosine triphosphate (ATP) from PmrB to PmrA (A) and QseB (B) in the absence of ferric iron. *n* ≥ 3. (**C** and **D**) Representative radiographs tracking the autophosphorylation of PmrB and the subsequent phosphotransfer of radiolabeled ATP from PmrB to PmrA (C) and QseB (D) in the presence of Fe^3+^. *n* ≥ 3. (**E** and **F**) Representative quantification of phosphorylated forms of the response regulators (RR∼P) PmrA (PmrA∼P, squares) and QseB (QseB∼P, triangles) over time in the absence (E) or presence (F) of Fe^3+^. Abundance was normalized relative to abundance at time 0.

The above experiments indicated a strong surge in *qseBC* transcript in response to ferric iron that only occurs when the QseBC and PmrAB systems are intact. These studies also suggested that PmrB and not QseC, where the UTI89Δ*qseC* strain did not undergo a transcriptional surge, likely mediates the response to ferric iron by phosphorylating both PmrA and QseB. We thus evaluated the kinase activity of PmrB toward PmrA and QseB, all isolated from UTI89, in the presence and absence of ferric iron (29). For our studies, we used membrane fractions enriched with PmrB from strain UTI89, which harbors 98% nucleotide identity and 99% protein sequence identity to previously tested, non-pathogenic *E. coli* strain K12 (table S1) (45). In the absence of signal, UPEC PmrB exhibited strong phosphatase activity toward PmrA isolated from UTI89 (Fig. 2A). This observation was consistent with previous reports evaluating PmrB activity in nonpathogenic *E. coli* (45). However, PmrB indiscriminately phosphorylated QseB, isolated from UTI89, in the absence of signal (Fig. 2B).

When the phosphotransfer assays were repeated with 100 μM ferric iron added to the reaction buffer, PmrB phosphotransfer to both PmrA and QseB increased (Fig. 2, C and D). On average, maximal phosphorylation of the response regulators was observed 10 min after addition of the stimulus, with the highest rate of phosphorylation occurring within the first 2 min of the reaction (Fig. 2, E and F). These data indicate that the presence of ferric iron changed the kinetic behavior of PmrB toward both cognate (PmrA) and noncognate (QseB) partners.

### QseB and PmrA cooperatively control the expression of ferric iron–regulated targets

**Fig. 3:**
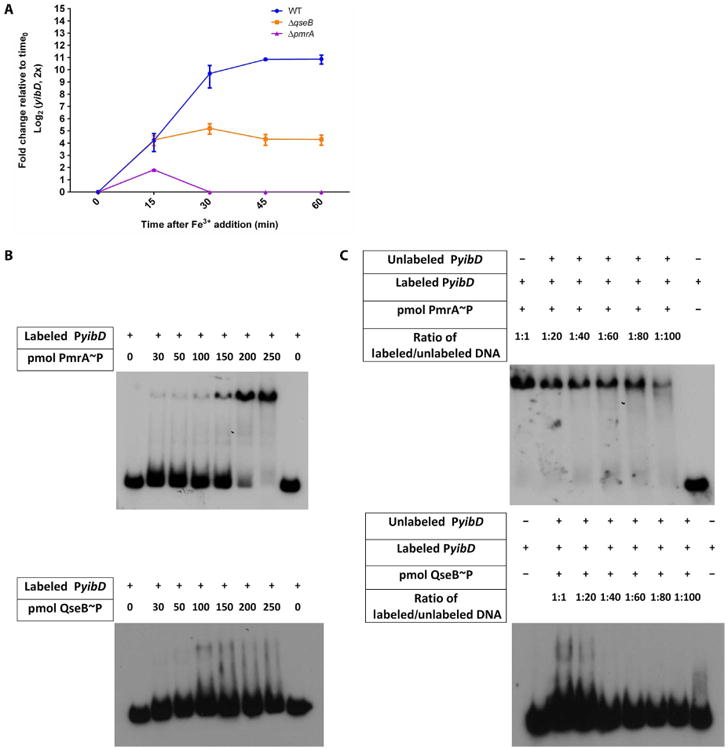
Additional targets directly controlled by both PmrA and QseB in response to ferric iron (**A**) *yibD* expression in response to Fe^3+^ was measured in UTI89, UTI89Δ*pmrA*, and UTI89Δ*qseB* using qRT-PCR. The abundance of *yibD* at each time point was normalized to the abundance in each strain at time 0, using *gyrB* as an endogenous control to calculate ΔΔC_T_ values. Error bars indicate SEM, *n* = 3. WT, wild type. (**B**) EMSA using a radiolabeled 105–base pair (bp) fragment of the *yibD* promoter incubated with indicated amounts of in vitro PmrA∼P or QseB∼P. (**C**) Mobility shift of radiolabeled *yibD* promoter induced by incubation with PmrA∼P or QseB∼P in the presence of increasing concentrations of unlabeled *yibD* promoter. Blots are representative of at least three biological replicates.

Given the activation of PmrA and QseB in response to ferric ironinvitro, we assessed whether PmrA and QseB were both required for optimal induction of ferric iron–stimulated transcripts. YibD is a glycosyltransferase, and *yibD* expression is stimulated by PmrA in response to ferric iron in *Salmonella enterica* (41, 46, 47). The promoter of UPEC *yibD* also contains a PmrA binding consensus site and is bound by PmrA in in vitro assays (23). Whereas ferric iron induced a surge of *yibD* expression in the UPEC strain UTI89, this surge was abolished in UTI89Δ*pmrA* and reduced by fivefold in UTI89 Δ*qseB* (Fig. 3A). Subsequent electrophoretic mobility shift assays (EMSAs) indicated that PmrA and QseB each bound to the *yibD* promoter, albeit with different binding affinities (Fig. 3, B and C). Addition of in vitro phosphorylated PmrA (PmrA∼P) caused a discernable mobility shift of DNA corresponding to a portion of the *yibD* promoter at a concentration of 30 pmol of purified protein per reaction, whereas in vitro QseB∼P (24) caused a mobility shift only when present at a concentration of 100 pmol per reaction (Fig. 3, B and C).

### PmrA and QseB mediate UPEC tolerance to PMB

**Fig. 4:**
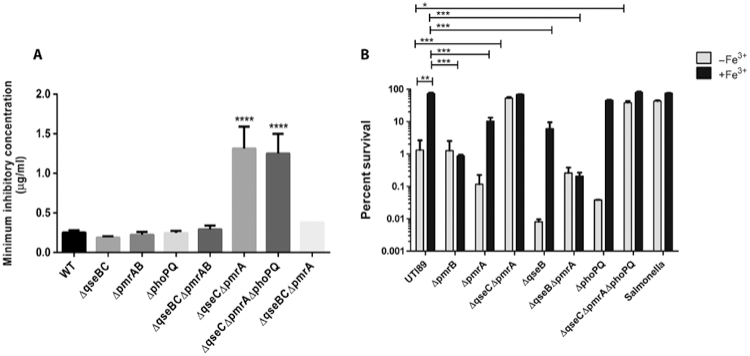
Ferric iron enhances resistance to PMB in a manner that depends on both PmrA and QseB (**A**) The MICs for PMB without pretreatment of bacteria with ferric iron were calculated for UTI89 and the indicated UTI89 deletion strains. Error bars represent SEM, *n* ≥ 4. Statistical analyses were performed using analysis of variance (ANOVA), ****P ≤ 0.0001. (**B**) Tolerance of UTI89, indicated UTI89 deletion strains, and *S. enterica* Typhimurium 14028 to PMB with or without Fe^3+^ preconditioning. Error bars represent SEM, *n* = 3; *P < 0.05, **P ≤ 0.01, ***P ≤ 0.001.

The PmrAB TCS induces LPS modifications to buffer against damage caused by antimicrobial peptides such as PMB (41). The PhoPQ TCS cooperates with PmrAB to control LPS modifications in response to increased ferric iron and decreased magnesium ions in *Salmonella* (30). However, the same coordination of the response to ions has not been demonstrated for PmrAB and PhoPQ in *E. coli* (48). On the basis of the observation that QseB and PmrA costimulate the expression of *yibD* (Fig. 3A), a target associated with LPS modifications, we tested the UPEC strain UTI89 and UTI89 mutants harboring deletions of QseBC, PmrAB, and PhoPQ components for PMB resistance. The minimum inhibitory concentration (MIC) of PMB was similar between wild type and mutants lacking both components of the QseBC, PmrAB, or PhoPQ TCSs (Fig. 4A), indicating an overall similar baseline susceptibility to PMB. However, UTI89 Δ*qseC* Δ*pmrA*, in which cross-interaction between PmrB and QseB is favored, exhibited a statistically significant increase in PMB tolerance (Fig. 4A). This increase in MIC observed in UTI89 Δ*qseC*Δ*pmrA* was unchanged by the additional deletion of phoPQ (Fig. 4A). However, when *qseB* was additionally deleted from UTI89Δ*qseC*Δ*pmrA*, the MIC returned to wild-type susceptibility concentrations.

Previous studies by Winfield and Groisman indicated that pre-treatment of *E. coli* with sublethal concentrations of ferric iron boosted tolerance to PMB (48). Given the increased tolerance of UTI89Δ*qseC*Δ*pmrA* to PMB (in which the absence of QseC and PmrA favors the interaction between PmrB and QseB) and the observed ability of PmrB to phosphorylate both PmrA and QseB in response to ferric iron, we asked whether pretreatment with ferric iron would prime UPEC to mitigate PMB-induced damage in a manner that depended on both PmrA and QseB. To test this, we grew the UPEC strain UTI89 and isogenic *pmr* and *qse* deletion mutants in the presence or absence of ferric iron for 2 hours and then exposed the cells to PMB (2.5 μg/ml) and subsequently assessed survival. As a control, *S. enterica* serovar Typhimurium strain 14028 was included in the experiments because it has previously been shown that *Salmonella* tolerance to PMB increases after incubation with ferric iron in a manner that is dependent on PmrAB and PhoPQ (48). Consistent with previous observations, *Salmonella* exhibited a higher overall tolerance to PMB, even in the absence of ferric iron conditioning. Conditioning with ferric iron before exposure to PMB resulted in comparable survival for the UPEC strain UTI89 and *Salmonella*, at about 75% (Fig. 4B). In UPEC, percent survival after preconditioning was statistically significantly decreased in the absence of PmrA, declining to 20%. Strikingly, the mutant lacking QseB exhibited even greater reduction in survival after PMB treatment despite ferric iron preconditioning, declining to about 10% compared to the pretreated UPEC strain UTI89. The mutants lacking both *pmrA* and *qseB* exhibited survival comparable to that of the Δ*pmrB* mutant. Unlike what has been reported for *Salmonella* (48, 49), deletion of *phoPQ* did not statistically significantly alter UTI89 survival, indicating that preconditioning with ferric iron increases UPEC tolerance to PMB through coordinated regulation of downstream targets by PmrAB and QseBC.

To determine whether this phenotype was strain-specific, we tested various other strains of extraintestinal pathogenic *E. coli* (ExPEC), including the well-characterized strains EC958 and CFT073, as well as urinary isolates collected from the Vanderbilt University Hospital. PMB tolerance after ferric iron preconditioning varied among the different strains. Vanderbilt urinary tract isolates (VUTIs) 39, 47, 61, and 77 and CFT073 exhibited increases in PMB tolerance after ferric iron pretreatment, with VUTI77 and CFT073 having the highest tolerance (fig. S3). However, VUTI61 and EC958 exhibited no difference in PMB susceptibility with or without ferric iron pretreatment, suggesting that the observed effects with PMB are neither strain-specific nor universally shared among all urinary *E. coli* isolates. Together, our analyses have uncovered a previously uncharacterized interaction between PmrA and QseB that mediates resistance to PMB in a subset of *E. coli* strains.

## Discussion

A handful of previous studies have described histidine kinases that are capable of phosphorylating both their cognate partner and a noncognate response regulator in wild-type bacterial cells (17, 19–21). These examples can be found in multiple bacterial species, including cross-phosphorylation of YycF by PhoR in *Bacillus subtilis* (18) and interactions between ArcB and OmpR in *E. coli* (17). In these examples, cross-regulation between TCSs is critical for mediating appropriate responses to environmental stress. However, in these cases, the presence of the two interacting noncognate partners is sufficient for the proper response, unlike the QseBC and PmrAB systems, where all four components are required for appropriate responses to signal (Fig. 1).

In *Rhodobacter capsulatus*, interacting TCSs NtrBC and NtrXY have been reported to mediate nitrogen responses. Bacteria lacking the NtrC response regulator, or both the NtrY and NtrB histidine kinases, cannot properly use molecular nitrogen (N_2_) or urea as a nitrogen source. This suggests interactions between noncognate partners NtrY and NtrC in wild-type cells (20). However, unlike the PmrB noncognate interaction described in this study, no specific signal that initiates NtrY-NtrC interactions has been identified. NarPQ and NarLX are interacting TCSs that control nitrate metabolism in the nonpathogenic *E. coli* strain K12. The histidine kinases NarQ and NarX can phosphorylate both response regulators NarL and NarP both during in vitro assays and under physiologic conditions within the bacterium. NarX preferentially senses nitrate, but NarQ senses both nitrate and nitrite. To fine-tune responses to these stimuli, there is a kinetic bias toward the different response regulators (19, 50). Whereas NarQ has a slight kinetic preference for NarL, NarX has a very strong kinetic preference for NarL, which allows NarX to de-phosphorylate NarL when nitrate is absent (19, 50, 51). In contrast, the QseBC-PmrAB interactions described here are mediated by a single stimulus, which culminates in the kinetically equivalent phosphorylation of two response regulators, at least based on in vitro phosphotransfer assays (Fig. 2).

Although our transcriptional studies focused mostly on the *qseBC* operon, we observed similar interactions for an additional shared transcriptional target, *yibD*. This target was previously reported to be part of the extensive PmrAB regulon in *Salmonella* (41, 46, 47). To date, the only transcriptional targets reported for QseB have been *qseBC* and *flhDC* (52, 53). Deletion of either *pmrA* or *qseB* diminished the *yibD* and *qseBC* transcriptional surge in response to ferric iron (Fig. 3A), suggesting that both *yibD* and the *qseBC* oper-on are part of the QseBC-PmrAB regulon in *E. coli* and that QseB augments transcription of *yibD* in the presence of PmrA. In other reports, mutation of *pmrA* decreases the survival of *E. coli* MG1655 in the presence of PMB by several orders of magnitude (48). Here, we show that the decrease in survival caused by PMB is smaller for UTI89 than that reported for MG1655, but this may be a result of pathotype differences between these strains. Differences in tolerance to antimicrobial agents are highly variable in the VUTI strains we analyzed; this may be due to single-nucleotide polymorphisms or other genomic variations in these strains, which we are currently investigating. As expected, deletion of *pmrB* abolished the *qseBC* transcriptional surge, consistent with PmrB being the sole ferric iron sensor transducing signals to QseB or PmrA, or both (Figs. 1 and 2).

The involvement of QseC in mediating the proper surge and decline of the transcriptional responses to ferric iron is not yet clear; when QseC is absent, any *qseBC* transcriptional surge is obscured by constitutively high *qseB* expression (Fig. 2E). One possible mechanism for QseC-mediated control of the ferric iron response is heterodimerization of QseC with PmrB, which would prevent aberrant phosphotransfer between PmrB and QseB until the presence of ferric iron favors the formation of homodimers. Alternatively, QseC could sequester QseB and prevent QseB from interacting with and being phosphorylated by PmrB in the absence of signal. Future studies will focus on delineating the potential protein-protein interactions that could be contributing to the tight control of QseBC-PmrAB responses to ferric iron.

**Fig. 5:**
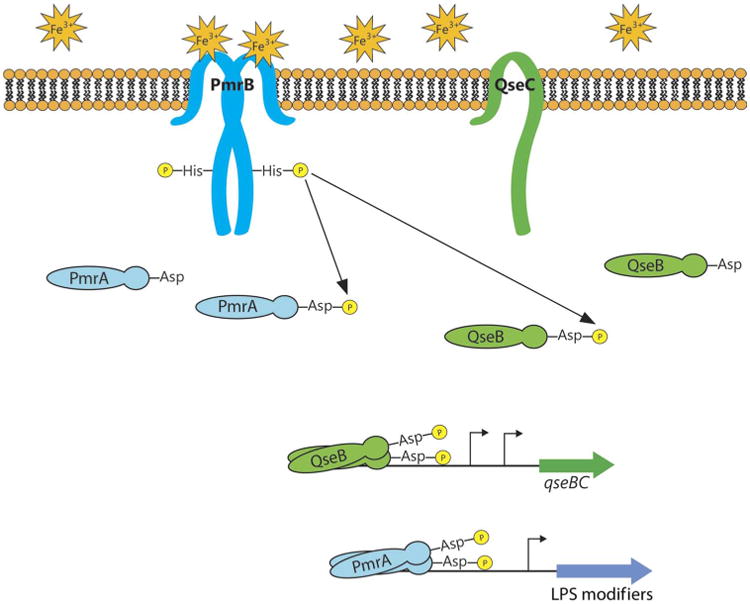
Model of PmrAB and QseBC signal transduction in response to ferric iron Ferric iron is sensed by the sensor kinase PmrB, which, in turn, phosphorylates both the cognate response regulator PmrA and the noncognate response regulator QseB. The phosphorylated response regulators stimulate transcription from the *qseBC* promoter and alter the expression of genes involved in modifying LPS. The role of QseC is ambiguous in the signaling cascade but is required for physiologically relevant signaling in the UPEC strain UTI89.

In silico sequence scanning reveals that PmrB and QseC share 33.53% sequence identity. Other sensors with similarly high identity, such as NarX and NarQ (28.5% identity) and AtoS and ZraS (27.6% identity), are known to cross-regulate to allow cells to adjust to environmental stress (20, 54). The increase in PMB tolerance seen in UTI89 after ferric iron conditioning (Fig. 5) suggests that the cross-interactions between QseBC and PmrAB may aid the bacteria in fine-tuning responses to stress imposed by cationic stress such as the last line of defense drug, colistin, which exhibits the same mechanism of action as PMB. For example, during acute urinary tract infection, bacteria are starved for iron (36, 55, 56), yet, at the same time, they must be able to distinguish between the metals that are required for cellular metabolism and detrimental cations and cationic polypeptides that are deployed by the innate immune response because they are catastrophic to bacterial membrane integrity. Increased tolerance to PMB was not specific to strain UTI89 because other ExPEC strains, especially VUTI77 and CFT073 (fig. S3), exhibited increased PMB tolerance after ferric iron preconditioning. Although the increase in survival was not as robust as that observed in UTI89, there could be differences in the extent of cross-interactions between the QseBC and PmrAB TCSs or the amino acid sequence differences in PmrAB and QseBC that may exist between these strains. For example, the enterohemorrhagic *E. coli* strain Sakai harbors a truncated QseC. Ongoing deep sequencing experiments probe the PmrAB-QseBC regulon in response to ferric iron, aiming to further elucidate the cationic response driven by cross-interactions between PmrAB and QseBC in UPEC.

## Materials and Methods

### Bacterial strains and growth conditions

All bacterial strains are listed in table S2. Cultures were grown in Lysogeny broth (Fisher) or N-minimal broth [described in (24)] with or without 100 μM ferric iron (Fisher) or 100 μM epinephrine (Sigma) at 37°C with shaking. UTI89Δ*qseC*, UTI89Δ*pmrB*Δ*qseC*, and the corresponding pQseC, pQseC-mycHis, pPmrB, and pQseC-mycHis plasmid constructs harboring the corresponding wild-type *qseC* and *pmrB* gene sequences were created previously (23, 24) and are listed in table S3. Promoter activity for the *qseBC* operon was measured using a previously constructed plasmid (23, 24), listed in table S3, in which the *qseBC* promoter region is fused to *gfp*.

### Phosphotransfer assays

Membranes enriched for UTI89-derived PmrB or QseC (7 μg) were incubated with purified QseB (14 μg) and 0.7 μCi [γ-^32^P]ATP, in the absence or presence of signal, in 1× tris-buffered saline (TBS), 0.5 mM dithiothreitol (DTT), and 0.5 mMMgCl_2_ per reaction. Aliquots (10 μl) were withdrawn from this reaction mix at different time points, mixed in a 1:1 ratio with 2× SDS loading buffer, and kept on ice until SDS– polyacrylamide gel electrophoresis (SDS-PAGE) analysis. Gels were dried and exposed to x-ray film for 48 hours at −80°C. Band intensities corresponding to QseB∼P over time were quantified using ImageJ software and normalized to QseB∼P at time = 0. All experiments were repeated two to four times.

### Phosphatase assays

Glutathione Sepharose beads (GE Healthcare Life Sciences) fused to the cytosolic portion of PmrB, as described previously (22), were prepared and used to in vitro phosphorylate QseB as described in (57). QseB∼P (0.2 nmol) was incubated at room temperature with 7 μg of membrane vesicles in the presence or absence of ferric iron with 1× TBS, 0.5 mM DTT, and 0.5 mM MgCl_2_. Aliquots (10 μl) were withdrawn from the reaction at different time points, mixed in a 1:1 ratio with 2× SDS loading buffer, and kept on ice until SDS-PAGE analysis. Gels were dried and exposed to x-ray film at −80°C. Band intensities corresponding to QseB∼P over time were quantified using ImageJ software and normalized to QseB∼P at time = 0.

### Purification of tagged QseB and PmrA

The pQseB-mycHisA and pPmrA-mycHisA plasmid constructs were used for expression and purification of tagged QseB and PmrA, respectively, were previously constructed (24). QseB or PmrA expression was induced with 0.1% arabinose, and the tagged proteins were affinity-purified using a TALON column (Clontech) followed by anion exchange chromatography through a Mono Q column (GE Healthcare), as described previously (24).

### Electrophoretic mobility shift assays

Purified QseB-mycHisA and PmrA-mycHisA were phosphorylated in vitro using glutathione Sepharose beads fused to the cytosolic portion of PmrB, as described previously (22). Phosphorylated QseB-mycHisA or PmrA-mycHisA (0 to 250 pmol per reaction) was incubated with about 6 fmol of a 105-bp fragment of the *yibD* promoter region in binding buffer (final concentration: 20 mM tris-HCl, 5 mM MgCl_2_, 5 mM KCl, 10% glycerol) for 20 min at room temperature. Reactions were loaded onto a 5% acrylamide nondenaturing gel, and electrophoresis was performed for 2.5 hours at 50 V. Gels were dried at 80°C for 2 hours before they were exposed to x-ray film at −80°C for 2 hours to overnight.

### qRT-PCR expression analysis

Cultures were grown to log phase at 37°C with shaking, and samples were collected at various time points and flash-frozen until RNA extraction. RNA was extracted using the RNeasy kit (Qiagen), deoxy-ribonuclease (DNase)–treated using TURBO DNase I (Ambion), and reverse-transcribed using SuperScript II Reverse Transcriptase (Invitrogen). DNase-treated RNA samples not subjected to reverse transcription were used as negative controls. Complementary DNA (cDNA) was amplified using the *gfp*- and *rrsH*-specific primers listed in table S4. qRT-PCR was performed, using an ABI StepOne Plus Real-Time PCR machine and multiplexed TaqMan MGB chemistry, in triplicate with two different amounts of cDNA (50 or 25 ng per reaction). Relative fold change was determined by the ΔΔC_T_ method where transcript abundances were normalized to *rrsH* abundance.

### PMB sensitivity assay

Bacteria were grown overnight at 37°C with shaking. Overnight cultures were then subcultured into N-minimal medium with or without 100 μM ferric chloride. Once N-minimal cultures reached mid-logarithmic phase of growth, cultures were normalized to an OD_600_ (optical density at 600 nm) of 0.3 in phosphate-buffered saline (PBS) and incubated with or without PMB (2.5 μg/ml) at 37°C for 1.5 hours. Cells were plated on LB agar to determine colony-forming units per milliliter. Percent survival was calculated by dividing the number of bacteria that grew after exposure to PMB by the number of bacteria that grew after incubation in PBS alone and multiplying the quotient by 100. The MICs for PMB without pretreatment of bacteria with ferric iron were calculated using Etest strips (BioMérieux).

### Statistics

All statistical analyses were performed using GraphPad Prism software. When calculating the survival ratio in the PMB sensitivity assay, preconditioned and nonconditioned bacteria were compared pairwise between strains and were shown to be statistically significant between the indicated strains using a nonparametric one-way ANOVA by the Kruskal-Wallis test, *P* < 0.01.

## Supplementary Material

Supplement Fig. S1. QseC activity is not enhanced in the presence of epinephrine.
Fig. S2. The *qseBC* transcriptional surge is specific to ferric iron.
Fig. S3. PMB tolerance after ferric iron preconditioning varies between clinical urinary isolates.
Table S1. QseB and QseC protein sequence identity among *E. coli* strains and other enteric bacteria.
Table S2. Bacterial strains.
Table S3. Plasmids.
Table S4. Primers and probes.
