# Supplementary material for: Signaling by two-component system noncognate partners promotes intrinsic tolerance to polymyxin B in uropathogenic Escherichia coli
Source: Sci Signal. Author manuscript; Available in PMC 2017 Nov 9. (PMC5677524; doi:10.1126/scisignal.aag1775)
Supplement: Supplement — Fig. S1. QseC activity is not enhanced in the presence of epinephrine. Fig. S2. The qseBC transcriptional surge is specific to ferric iron. Fig. S3. PMB tolerance after ferric iron preconditioning varies between clinical urinary isolates. Table S1. QseB and QseC protein sequence identity among E. coli strains and other enteric bacteria. Table S2. Bacterial strains. Table S3. Plasmids. Table S4. Primers and probes. [file NIHMS911458-supplement-Supplement.pdf]

Science Signaling
AAAS

www.sciencesignaling.org/cgi/content/full/10/461/eaag1775/DC1

Supplementary Materials for

Signaling by two-component system noncognate partners promotes intrinsic tolerance to polymyxin B in uropathogenic *Escherichia coli*

Kirsten R. Guckes, Erin J. Breland, Ellisa W. Zhang, Sarah C. Hanks, Navleen K. Gill, Holly M. S. Algood, Jonathan E. Schmitz, Charles W. Stratton, Maria Hadjifrangiskou*

*Corresponding author. Email: maria.hadjifrangiskou@vanderbilt.edu

Published 10 January 2017, *Sci. Signal.* **10**, eaag1775 (2017)
DOI: 10.1126/scisignal.aag1775

This PDF file includes:

Fig. S1. QseC activity is not enhanced in the presence of epinephrine.
Fig. S2. The *qseBC* transcriptional surge is specific to ferric iron.
Fig. S3. PMB tolerance after ferric iron preconditioning varies between clinical urinary isolates.
Table S1. QseB and QseC protein sequence identity among *E. coli* strains and other enteric bacteria.
Table S2. Bacterial strains.
Table S3. Plasmids.
Table S4. Primers and probes.

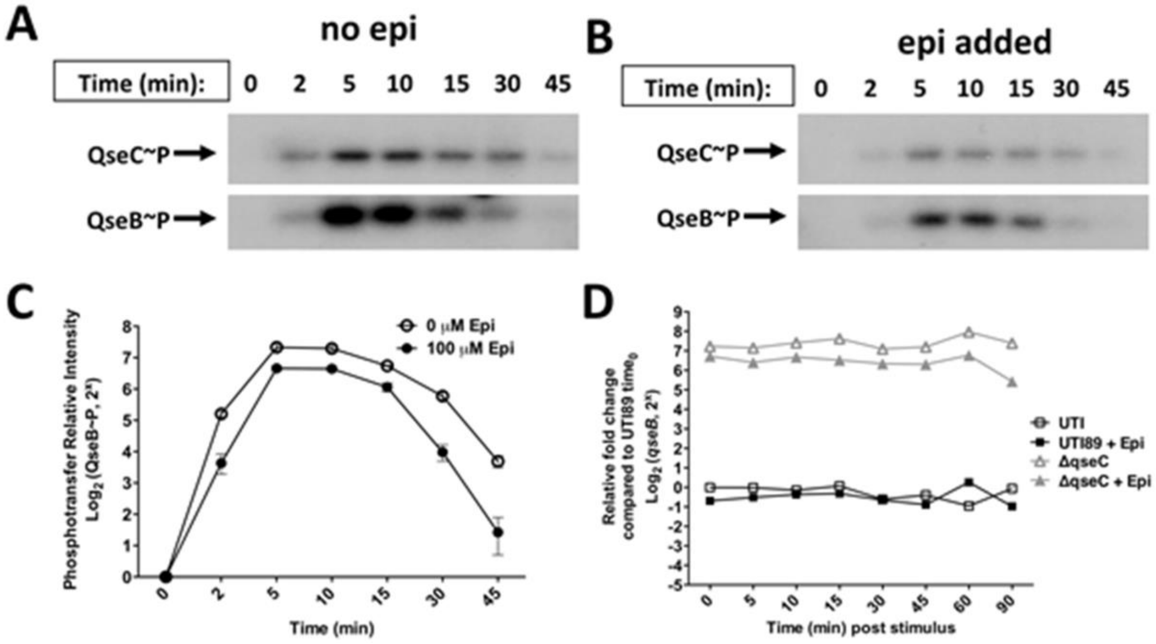

Fig. S1. QseC activity is not enhanced in the presence of epinephrine.
(A-B) Panels show radiographs that track autophosphorylation and subsequent phosphotransfer of ^{32}P-γATP to QseB by QseC in UTI89 membrane fractions in the absence (**A**) and presence (**B**) of epinephrine (Epi). N≥3 biological replicates. (**C**) Representative quantification of phosphorylated QseB (QseB~P) in the presence (filled circle) or absence (open circle) of epinephrine using image J. (**D**) Representative qRT-PCR analysis tracking the relative fold change of *qseB* transcript in wild-type UTI89 (squares) and UTI89Δ*qseC* (triangles) in the presence (filled shape) or absence (open shape) of epinephrine. Fold changes are graphed on a Log_2 scale. N=2

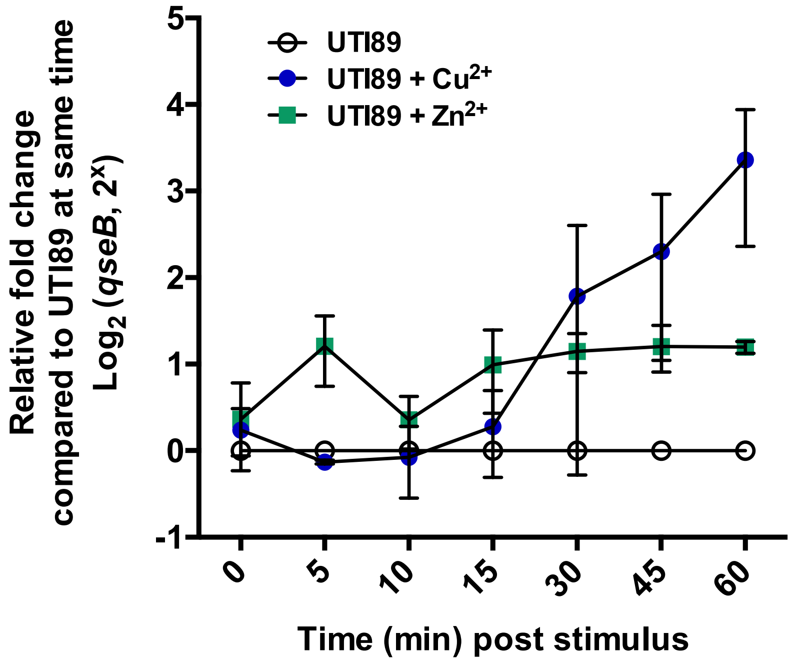

Fig. S2. The *qseBC* transcriptional surge is specific to ferric iron.
The graph depicts qRT-PCR analysis of *qseB* transcript abundance in UTI89 in N-minimal medium (open circles), UTI89 in the presence of copper (blue circles), and UTI89 in the presence of zinc (green squares). Fold changes were calculated using the ΔΔCT method, where *rrsH* was used as an endogenous control and samples were normalized to matching time points of UTI89 in the absence of added metal cations. Fold changes are graphed on a Log_2 scale. Error bars indicate standard error of the mean (SEM), N=3 biological replicates.

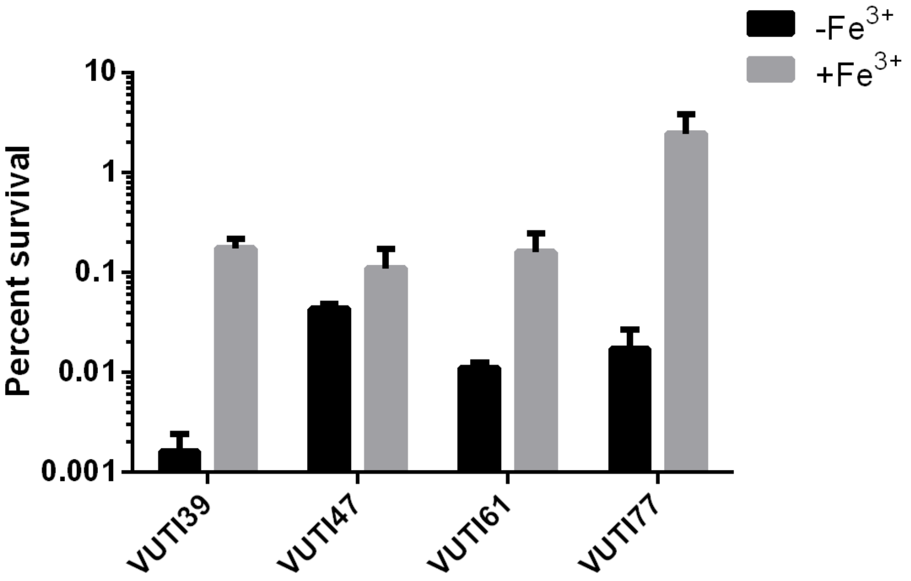

Fig. S3. PMB tolerance after ferric iron preconditioning varies between clinical urinary isolates.

The graph depicts tolerance of various clinically isolated *E. coli* strains to 2.5 µg/mL polymyxin B with or without ferric iron preconditioning. "$-Fe^{3+}$" indicates cells grown in N-minimal media without additional ferric iron before exposure to polymyxin B. "$+Fe^{3+}$" indicates cells grown in N-minimal media with ferric iron before exposure to polymyxin B. Survival was calculated by dividing the number of colony forming units (CFUs) recovered after polymyxin B incubation by the number of CFUs recovered after incubation in PBS alone, and multiplying by 100. Error bars represent standard error of the mean (SEM), N=3 biological replicates.

Table S1. QseB and QseC protein sequence identity among *E. coli* strains and other enteric bacteria.

Clustal Omega was used to align and compare sequence. The percent identity was reported from the percent identity matrix following alignment.

Strain	QseB protein sequence identity (%)	GenBank accession number	QseC protein sequence identity (%)	GenBank accession number
E. coli				
**UPEC str. UTI89**	100	ABE08897.1	100	ABE08898.1
**APEC O1K1 str. O1**	100.00	ABJ02530.1	100.00	ABJ02531.1
**B2 phylogenetic group: O83:H1**	100.00	YP_006121348.1	98.89	YP_006121349.1
**D1 phylogenetic group: UMN026**	100.00	YP_002414171.1	98.89	YP_002414172.1
**D2 phylogenetic group: IAI39**	100.00	YP_002409426.1	98.22	YP_002409427.1
**UPEC str. CFT073**	99.54	AAN82208.1	98.89	AAN82209.1
**K12 str. MG1655**	99.54	NP_417497.1	98.89	NP_417498.1
**B1 phylogenetic group: O104:H4**	99.54	AFS72693.1	98.22	AFS72692.1
**EAEC str. E55989**	99.54	CAU99558.1	98.22	CAU99560.
**EHEC str. EDL933**	99.54	AIG70396.1	98.22	AIG70397.1
**EPEC O55:H7 str. CB9615**	99.54	ADD58237.1	98.22	ADD58238.1
**O157:H7 str. Sakai Sakai***	99.54	NP_311934.1	97.13 / 98.55	NP_3909913-3910437<br>NP_3910431-3911261
**ETEC O139:H28 str. E24377A**	99.07	ABV19769.1	98.22	ABV17955.1
**Additional enteric bacteria**				

Shigella sonnei **str. Mosely**	100.00	EJL13232.1	99.11	EJL13233.1
Salmonella enterica **Typhumurium str. LT2**	87.67	NP_462092.1	79.29	NP_462093.1
Salmonella enterica **Typhumurium str. 14028S**	87.67	ACY90252.1	79.29	ACY90253.1
Klebsiella pneumoniae **str. 342**	83.11	ACI08570.1	67.04	ACI08526.1
Edwardsiella tarda	74.89	ADO13165.1	56.35	ADO24152.1

***Sakai has a stop codon in the middle of this putative QseC sequence rendering QseC non-functional in this strain.**

Table S2. Bacterial strains.

Strain	Source
UTI89	Mulvey *et al*. (41)
UTI89Δ*qseC*	Kostakioti *et al.* (24)
UTI89Δ*pmrB*	Guckes *et al*. (23)
UTI89Δ*qseB*	Kostakioti *et al.* (24)
UTI89Δ*pmrA*	Guckes *et al*. (23)
UTI89Δ*pmrAB*	Guckes *et al*. (23)
UTI89Δ*qseBC*	Kostakioti *et al.* (24)
UTI89Δ*qseB*Δ*pmrA*	This study
UTI89Δ*qseC*Δ*pmrA*	Guckes *et al*. (23)
UTI89Δ*qseBC*Δ*pmrA*	This study
UTI89Δ*phoPQ*	This study
UTI89Δ*qseC*Δ*pmrA*Δ*phoPQ*	This study
Salmonella enterica Typhimurium 14028	Mark Goulian

Table S3. Plasmids.

Plasmid	Source
pTrc99A_pQseC	(24)
pTrc99A_pPmrB	(24)
pBADmycHisA_PmrA	(23)
pBADmycHisA_QseB	(24)
pBADMycHisA_PmrB	(23)
pBADmycHisA_QseC	(23)
Pqse::gfp	(23)

Table S4. Primers and probes.

Primer/Probe	Nucleotide Sequence (5'→3')	Purpose
rrsH483_Fwd	CGTTACCCGCAGAAGAAGCAC	qRT-PCR
rrsH637_Rev	GATGCAGTTCCCAGGTTGAGC	qRT-PCR
rrSH probe	VIC-CGTTAATCGGAATTACTG	qRT-PCR
gfp_qrt_Fwd	GTGCCATGCCCGAAGGTTATGTAC	qRT-PCR
gfp_qrt_Rev	GTTGTATTCCAATTTGTGTCCAAGAAT	qRT-PCR
gfp probe	FAM-ACGTGCTGAAGTCAAG	qRT-PCR
gyrB_qrt_Fwd	GATGCGCGTGAAGGCCTGATTG	qRT-PCR
gyrB_qrt_Rev	CACGGGCACGGGCAGCATC	qRT-PCR
gyrB probe	VIC-ACGAACTGCTGGCGGA	qRT-PCR
yibD_qrt_Fwd	GGTTCAACGGATAATTCTGTT	qRT-PCR
yibD_qrt_Rev	ACTTCAATCCCACGATTACG	qRT-PCR
yibD probe	NED-CACGTTCGTTTGTTGCATC	qRT-PCR
